# Impact of a pharmacist adherence intervention on medication beliefs and drug-related problems in coronary patients – a process evaluation of the MIMeRiC trial

**DOI:** 10.1186/s12913-026-15037-5

**Published:** 2026-09-15

**Authors:** Malin Johansson Östbring, Ahmed Al Musawi, Tommy Eriksson, Lina Hellström

**Affiliations:** 1 Pharmaceutical Department, Kalmar County Council, County Council Hospital, Building 2 Floor 2, Kalmar, 391 85 Sweden; 2 Department of Medicine and Optometry, Faculty of Health and Life Sciences, Linnaeus University, Kalmar, 391 82 Sweden; 3 Department of Biomedical Science and Biofilm – Research Center for Biointerfaces, Faculty of Health and Society, Malmö University, Jan Waldenströms gata 25, Malmö, 205 06 Sweden

**Keywords:** Complex interventions, Pharmaceutical care, Coronary artery disease, Drug-related problems, Drug-related side effects and adverse reactions, Quality of prescribing, Beliefs about medicines, Medication review, Motivational interviewing, Medication adherence

## Abstract

**Background:**

The Motivational Interviewing and Medication Review in Coronary Heart Disease (MIMeRiC) randomized controlled trial investigated the effects of a pharmacist-led intervention at a cardiology clinic on patient adherence and clinical outcomes. The results demonstrated that the intervention group had increased adherence to cholesterol-lowering medications and aspirin, as well as improved overall beliefs about medicines. However, the intervention did not lead to a higher proportion of patients reaching the target for low-density lipoprotein cholesterol (LDL-C) levels. This paper reports on the implementation of the intervention and explores potential mechanisms underlying these outcomes, focusing on the content of medication reviews, quality of prescribing, and changes in beliefs about medicines.

**Methods:**

Medication reviews were evaluated by classification of identified drug-related problems (DRPs), actions taken to address these problems, and the outcomes of those actions, utilizing data from electronic health records (EHR). The Medication Assessment Tool for secondary prevention of Coronary Heart Disease (MAT-CHDsp) was applied to EHR data to evaluate prescribing quality. Beliefs about medicines were evaluated using the Beliefs about Medicines Questionnaire specific (BMQ-S), completed by participants at baseline and follow-up. Descriptive and comparative statistics were used to analyze the data.

**Results:**

Medication reviews were conducted for 144 of the 159 patients in the intervention group, with a mean of 4.3 DRPs per patient. Pharmacists identified 500 DRPs and managed 245 of these with patients alone, while 196 required discussions with physicians. The resulting status of 218 DRPs was known, and for 194 of these, the issue was solved or improved. No differences in prescribing quality were observed between groups at six months post-discharge. The mean concern score on the BMQ-S decreased more in the intervention group, and 52.8% of intervention patients vs. 31.0% of control patients (*p* = 0.051) shifted from an ambivalent to an accepting attitudinal category.

**Conclusions:**

This process evaluation demonstrates that clinical pharmacists were effective in identifying and managing DRPs through collaboration with patients and physicians, leading to the resolution or improvement of a substantial number of DRPs. The intervention did not impact prescription quality. Patients in the intervention group reported reduced concerns about their medications, which explains the improved medication adherence.

**Trial registration:**

This study is based on data from a randomized controlled trial (RCT) registered at ClinicalTrials.gov (NCT02102503; registered 03/04/2014).

**Supplementary Information:**

The online version contains supplementary material available at https://doi.org/10.1186/s12913-026-15037-5.

## Background

Patients with coronary heart disease (CHD) who are non-adherent to their preventive medications face a higher risk of new cardiac events and mortality [1–4]. Consequently, improving adherence to these medications has become a critical issue in secondary prevention [5]. Simple as well as multifaceted interventions have been tested to improve patients’ adherence, but there is conflicting evidence regarding the most effective approach [6–8].

Many adherence interventions are classified as complex healthcare interventions. A complex intervention is defined as one that involves multiple components that interact or work independently or engage several stakeholders [9, 10]. Trials of complex interventions are sensitive to context, the characteristics of the targeted population, and the level of implementation of intervention activities. Process evaluations complement the outcomes evaluation and may include both prespecified questions grounded in the theory used to develop the intervention, as well as questions arising from hypotheses generated during the interpretation of the outcomes.

We conducted the Motivational Interviewing and Medication Review in Coronary Heart Disease (MIMeRiC) randomized controlled trial (RCT) using a complex intervention aimed at improving adherence and treatment among patients with CHD [11, 12]. The intervention, based on the concept of pharmaceutical care, focused on supporting patients’ beliefs about medicines and addressing drug-related problems (DRPs) during the first year following the diagnosis of CHD. The aim was to meet patients’ all drug-related needs and increase adherence to secondary preventive medications, thereby improving health outcomes.

The trial showed that the intervention led to increased adherence to cholesterol-lowering drugs (statins and/or ezetimibe) and aspirin at 15 months. In the intervention group, 87.8% of patients continued to refill their lipid-lowering prescriptions and reported that they used it, compared to 77.4% in the control group (*p* = 0.033). Similarly, adherence to aspirin was higher in the intervention group, with 97.1% refilling their prescriptions, compared to 91.2% in the control group (*p* = 0.036). However, the intervention did not increase the proportion of patients achieving the treatment target for low-density lipoprotein cholesterol (LDL-C) (< 1,8 mmol/L) after one year.

With the outcomes evaluation, we briefly reported on one process measure: patients’ beliefs about medicines. The intervention improved patients’ beliefs about their cardiac medicines, with intervention patients exhibiting a more positive necessity-concerns differential: 7.9 (5.7) vs. 6.3 (5.8) (*p* = 0.022). However, no differences between the groups were found in the proportion of patients categorized as accepting, ambivalent, indifferent, or skeptical [11]. A more detailed investigation is needed to understand how the intervention affected patients’ beliefs about medicines, as well as to assess measures of intervention implementation in order to interpret the findings of the MIMeRiC trial.

**Fig. 1:**
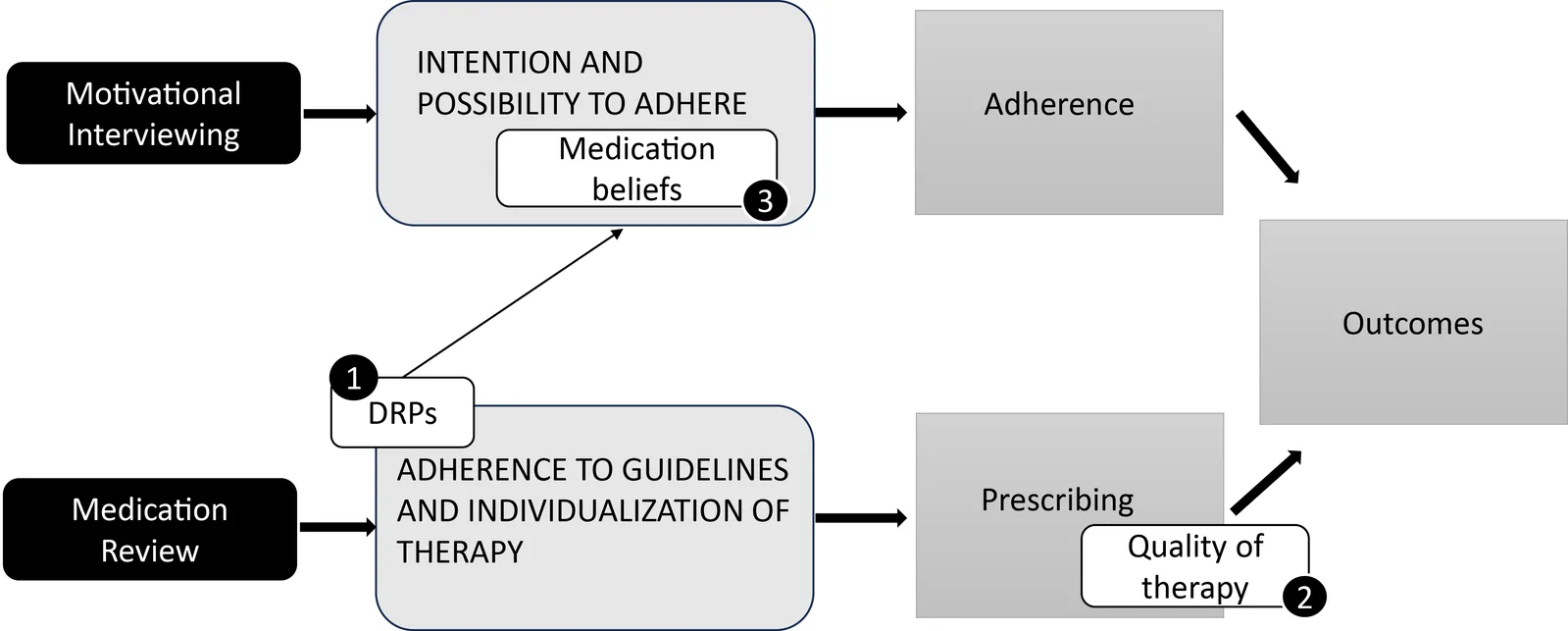
Overview of different components of MIMeRiC and corresponding objectives in this process evaluation. Activities (black), influencing factors (grey), sub-factors (white), and outcomes (grey squares) of the intervention in MIMeRiC, along with the objectives (black bullets) outlined in this process evaluation paper. The numbered objectives correspond to the following research questions in the study protocol for the process evaluation [13]: 1 = research question 1, 2 = research question 7, 3 = research question 5. DRPs = Drug-related problems

The pre-specified research questions for the full process evaluation of MIMeRiC are outlined in the study protocol [13]. This paper presents the first report from the process analysis, addressing three pre-specified research questions, as shown in Fig. 1: (1) What was actually delivered in the medication reviews? (2) Did the intervention change the quality of prescribing? (3) How did the intervention change patients’ beliefs about medicines? Additionally, based on questions raised during outcomes evaluation, we hypothesize that pharmacists and cardiologists may, in some cases, have opted for less intensive cholesterol-lowering treatment, assuming that fewer side-effects would promote higher long-term adherence. Therefore, we also investigate DRPs associated with cholesterol-lowering drugs in this study.

## Methods

This study is a process evaluation of the MIMeRiC randomized controlled trial. The study design, intervention, population, and sample size calculation have been described in detail elsewhere [11, 12]. Briefly, in MIMeRiC, patients were randomized to either a control group receiving standard care or an intervention group receiving standard care along with a clinical pharmacist-led follow-up program, which included a medication review and motivational interviewing (MI). The intervention started after the standard follow-up with nurses and physicians, which typically ended after 3–4 months. Patients in the intervention group participated in two in-person appointments and at least one follow-up phone call with LH or MJÖ, clinical pharmacists trained in MI and medication reviews. The intervention was tailored to each patient’s needs, with half of the intervention patients receiving 1–4 additional contacts, commonly by phone [11]. The clinical pharmacists were trained in medication review and MI and were evaluated as having “beginning proficiency” (≥ 3.5) in the global rating of MI-spirit. For a more detailed description, we refer to the MIMeRiC study protocol [9]. Patients in MIMeRiC had angiographically verified CHD and were recruited upon discharge or at the outpatient clinic at the County Hospital in Kalmar, Sweden, between 2013 and 2017. Data were collected from the Electronic Health Record (EHR) and patient questionnaires at baseline and at the 15-month follow-up.

This process evaluation of the MIMeRiC trial utilizes a combination of descriptive and comparative methods, depending on the specific research question. An overview of the methods is provided in Table 1, with further details below.

**Table 1:** Overview of methods

Research questions	Data	Source	Patients	Statistics
1. What was delivered in the medication reviews?	Documented DRPs, actions taken, documented results of changes	Pharmacists´ notes, EHR	Intervention group	Descriptive quantitative
2. Did the intervention change the quality of prescribing?	MAT-CHDsp at 6 months	EHR	Intervention group and control group	Comparative quantitative
3. How did the intervention change the patients’ beliefs about medicines?	Beliefs at baseline and follow-up	BMQ-Specific, paper questionnaire returned by participants	Intervention group and control group	Comparative quantitative

Abbreviations: DRPs, Drug related problems; EHR, Electronic Health Record; BMQ-Specific, Beliefs about Medicines Questionnaire Specific; MAT-CHDsp Medication Assessment Tool for evaluation of secondary prevention of Coronary heart Disease

### Medication reviews

During the medication reviews, pharmacists documented DRPs and the actions taken to handle them. A DRP was defined as “an event or circumstance that actually or potentially interferes with desired health outcomes” [14]. The number and type of DRPs were classified using the seven categories proposed by Cipolle et al. [15], with an additional “other” category that includes inconsistencies in medication lists. Two new categories were introduced to reflect the theoretical basis of the intervention: expressing negative beliefs about medicines and expressing a desire for more information about medicines. The actions taken by pharmacists and physicians in response to these DRPs, as well as any documented results, were also described (see supplement). In addition, we report on DRPs specifically related to lipid-lowering drugs and provide selected patient cases to give the reader a better understanding of the medication review process.

### Quality of prescribing

The Medication Assessment Tool for evaluation of secondary prevention of CHD (MAT-CHDsp) [16] is a clinical audit tool that summarizes review criteria based on clinical guidelines. The MAT-CHDsp was updated for use in this study and includes 23 criteria: 15 prescribing criteria (e.g., ”Patient with CHD is prescribed a daily low dose aspirin (75–100 mg)”) and eight follow-up criteria (e.g., “Patient with CHD and maintained on lipid lowering therapy … has achieved cholesterol levels of LDL cholesterol ≤ 1,8 mmol/L or > 50% reduction from initial value”). Adherence to quality criteria was considered high if > 80% of patients were prescribed the therapy or achieved therapy goals [17]. The MAT-CHDsp was used to assess the quality of treatment for all study patients six months after inclusion. The quality of treatment was compared between the intervention and control groups in terms of adherence and justified nonadherence to applicable criteria vs. non-adherence. A detailed description of the methods is provided in the supplement.

### Beliefs about medicines

Beliefs about Medicines Questionnaire-Specific (BMQ-S) [18] was administered to all study participants at baseline, 10 months, and follow-up. Participants were instructed to consider only their heart medications when answering the BMQ-S questions. Previously reported results from the BMQ-S are now complemented with data on changes in the sum of each subscale, changes in each item of the scales, and differences between groups in each item of the scale at follow-up. Additionally, we report how patients shifted to or maintained an accepting attitude during the study period, with comparisons between the intervention and control groups. Due to high rates of missing data at the 10-month follow-up (32.9% in the intervention group and 25.6% in the control group), we focused on reporting changes from baseline to follow-up. Temporal changes are reported for all patients in the control group who completed the BMQ-S at follow-up (*n* = 134), while in the intervention group, we report data only from patients who had follow-up data and were defined per protocol (*n* = 108). A detailed description of the methods is presented in the supplement.

### Statistical analysis

Analyses of the BMQ-S included an evaluation of the internal consistency of the necessity and concerns subscales at baseline and follow-up for the intervention and control groups, using Cronbach’s alpha coefficient. Group differences in the change in each scale score from baseline to follow-up were assessed using an independent t-test, with adjustments for sex, age, type of CHD, and history of CHD through linear regression. Within-group changes in each item of the scales were analyzed using a paired t-test, while differences between groups in individual items at follow-up were assessed using an independent t-test [11]. A chi-square test was used to compare accepting and ambivalent patients. The chi-square test was used to assess differences between intervention and control groups in MAT-CHDsp, while Fischer’s exact test was applied when the assumptions for the chi-square test were not met. All statistical analyses were conducted using IBM SPSS 24.0.

## Results

### Actions and results of medication reviews

Of the 159 patients randomized to the intervention group, 144 had at least one medication review. Among these patients, 138 (96%) had at least one DRP, with an average of 4.3 DRPs per patient (median: 3, range: 0–9). Pharmacists were able to identify DRPs during any contact with patients throughout the intervention period. A total of 553 contacts were registered for various purposes [11], including conducting medication reviews, follow-up of medication review outcomes, evaluating treatment changes, and performing MI.

In total, 500 DRPs were identified (Table 2). The most frequent DRP was *adverse drug reaction* (*n* = 154), reported in 96 patients (67%). Other common DRPs included *dose too low* (*n* = 66), *insufficient knowledge of purpose and/or function of drug* (*n* = 62), *nonadherence* (*n* = 51) and *need for additional drug* (*n* = 42). The majority of DRPs (*n* = 374, 75%) were related to drug treatment for cardiovascular disease.

**Table 2:** Identified drug-related problems (DRPs)

DRP type	Number of patients with DRPs (prevalence) *n* = 144	Number of DRPs identified for each type (percentage of total number of DRPs)	Number of DRPs related to CVD treatment identified for each type (percentage of all DRPs of this type)
Adverse drug reaction	96 (67%)	154 (31%)	124 (81%)
Patients expressing a desire for more information about medicines*	55 (38%)	62 (12%)	52 (84%)
Dosage too low	50 (35%)	66 (13%)	48 (73%)
Nonadherence	39 (27%)	51 (10%)	33 (65%)
Need for additional drug	35 (26%)	42 (8%)	26 (62%)
Patients expressing negative beliefs*	34 (24%)	34 (7%)	34 (100%)
Ineffective drug	24 (17%)	25 (5%)	15 (60%)
Dosage too high	22 (15%)	29 (6%)	23 (79%)
Unnecessary drug therapy	19 (13%)	22 (4%)	10 (45%)
Other*	15 (10%)	15 (3%)	8 (53%)
All		500 (100%)	374 (75%)

Abbreviation: CVD, Cardiovascular Disease

*DRP categories not described by Cipolle

**Fig. 2:**
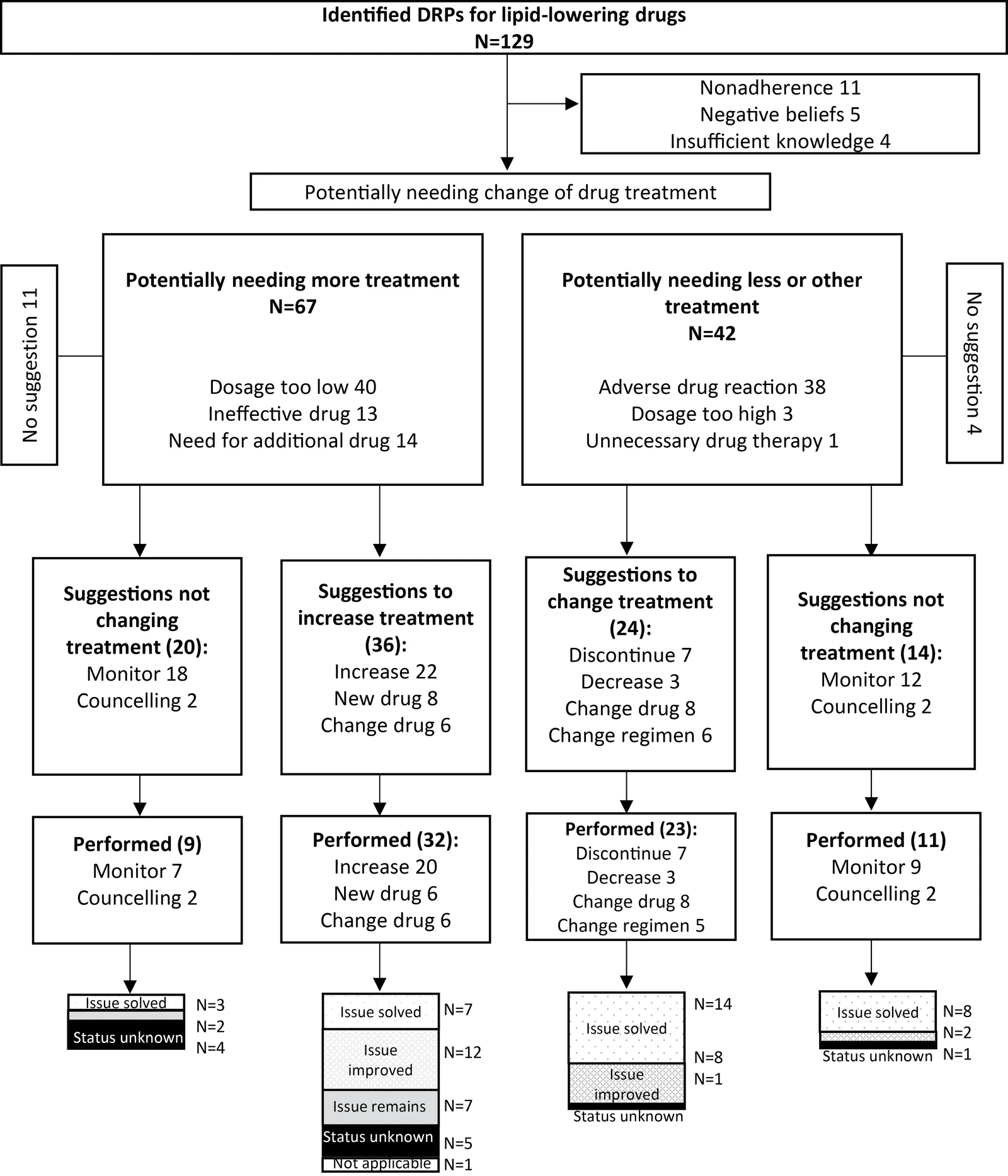
Flow chart of DRPs handled in the medication reviews. The medication review and treatment follow-up process was continuous, beginning with the identification of DRPs, represented at the center of the chart. DRPs were handled either through direct actions by the pharmacist in collaboration with the patient (right) or through suggestions made by the pharmacist to the physician (left). The bar charts on the left and right sides illustrate the outcomes of the medication reviews for each identified DRP. Abbreviation: DRP, Drug-related problem

Pharmacists handled DRPs through direct actions in collaboration with patients (*n* = 245) or by discussing issues with or providing suggestions to the involved physician (*n* = 196), as illustrated in Fig. 2. The most common response to DRPs involved providing patient-specific information or counselling by pharmacist (*n* = 169). Other frequent responses included suggestions to monitor or evaluate the drug treatment (*n* = 88) and suggestions to the physician to discontinue (*n* = 46) or change (*n* = 46) a medication (further details are provided in Supplementary Table S2). Of the suggestions communicated to prescribers (*n* = 196), the majority (*n* = 162, 83%) were accepted. There was no difference in acceptance rates between suggestions conveyed orally, via messages, or through referrals (Supplementary Table S3).

The outcomes of medication reviews, specifically how patients or DRPs were affected by treatment adjustments or other actions, are summarized in Table 3. For 186 DRPs, treatment adjustments were implemented, and resulting status was known for 147 cases. Among these, the issue was solved or improved in 135 cases. Additionally, 126 DRPs were managed through the provision of information or evaluation of drug treatment, with resulting status known for 71 DRPs. Of these, 59 issues were solved or status improved.

**Table 3:** Overview of actions and outcome of medication reviews

DRPs identified	Suggestions to handle DRPs	Actions by pharmacists or prescribers			Outcome			
					solved	improved	remained	unknown
*n*	*n*	*n*	Type of action	*n*	*n* (%)	*n* (%)	*n* (%)	*n* (%)
500	441	407	**Adjustment of therapy**	186	72 (39)	63 (34)	12 (6)	39 (21)
			**Information and/or evaluation**	126	41 (33)	18 (14)	12 (10)	55 (44)
			**Counselling by pharmacist**	95	Not investigated*			

Abbreviations: DRP, Drug related problem

*Resulting status not investigated for “Insufficient knowledge” or “Negative beliefs”, see supplement

### DRPs related to lipid-lowering drugs and patient cases illustrating medication reviews

Figure S2 in the supplement provides a flow chart detailing how DRPs related to lipid-lowering drugs were handled in the medication reviews. Among 129 identified DRPs, 67 were associated with a need for more treatment. Pharmacists suggested increased treatment in 36 cases, resulting in 32 adjustments being performed. However, 35 cases of LDL-C levels above target remained unchanged. In the majority of these cases (*n* = 31), pharmacists opted to accept the LDL-C value or monitor the treatment, in some cases because the patient was already on the maximum recommended dose of atorvastatin (80 mg). Of the 42 DRPs related to a potential need for reduced or change of treatment, the most common issue was adverse drug reactions. Treatment reductions were made in 10 cases. For nine adverse drug reaction cases, the suggestion was to monitor by pausing treatment and conducting follow-up assessments. This approach resolved the issue in eight cases, allowing patients to continue their lipid-lowering drug. In total, pharmacists’ actions resulted in 10 treatment reductions, 32 treatment increases, and 35 cases where treatment remained unchanged despite LDL-C levels being above target. Overall, most actions for lipid-lowering drugs led to the resolution or improvement of the identified DRPs.

A summary of nine patient cases is presented in Supplementary Table S4, illustrating the variety of DRPs related to lipid-lowering treatment and explaining some of the reasons why LDL-C above target was sometimes accepted. The table also includes corresponding data from the MIMeRiC trial on beliefs about medicines, LDL-C levels, and adherence. Additionally, Box 4 provides a detailed description of three patient cases from Table S4 to offer a richer understanding of how the medication reviews were conducted. These cases include a patient experiencing suspected statin side effects, a patient whose statin potency was successfully increased, and a patient facing multiple adherence barriers. All patient names used in these cases are fictitious.

**Table 4:** Three patient cases illustrating medication reviews

Anita, a 74-year-old woman with unstable angina, for which she has received a drug-eluting stent. She has a history of myocardial infarction 20 years ago, multiple percutaneous interventions, and atrial fibrillation. At discharge, her treatment was switched from simvastatin to atorvastatin 40 mg, lowering her LDL-C from 3.6 to 2.1 mmol/L. Before her follow-up with the pharmacist four months later, her atorvastatin dose was halved due to suspected stomach discomfort, although she felt this was related to another issue. The pharmacist explained the importance of maintaining a higher dose, and Anita agreed to try it. A week later, she reported increased stomach issues and flatulence, suspecting atorvastatin or ticagrelor (anti-platelet). After pausing atorvastatin for two weeks, she experienced worsened symptoms, prompting a resumption of the 40 mg dose after ruling it out as a cause. She was advised to consult her general practitioner about her stomach. At the ten-month follow-up, Anita continued atorvastatin and ticagrelor, with her LDL-C at a satisfactory 1.8 mmol/L. She reported having occasional stomach upset and expressed concern about the long-term effects of her medications but acknowledged her need for them.
Peter, a 69-year-old man with chronic coronary syndrome who had been treated with drug-eluting stents and had a history of deep vein thromboses managed with warfarin, received clopidogrel, metoprolol, and simvastatin 20 mg at discharge. His LDL-C decreased from 5.7 to 3.9 mmol/L, leading to an increase in simvastatin dosage to 40 mg during standard follow-up. Five months post-discharge, he reported feeling well but sometimes forgot to take his simvastatin, in his ambition to take it as late as possible. The pharmacist emphasized the need for a high-potency statin to control LDL-C levels and assisted Peter in establishing a realistic nighttime routine for taking simvastatin. Peter was open to treatment adjustments, and they agreed to recheck his LDL-C alongside his warfarin test. In the consultation, he also talked about his need to start exercise and change his meals. The pharmacist uses motivational interviewing, and they decide to follow up by phone. Two weeks later, Peter’s LDL-C was 3.2 mmol/L on 40 mg of simvastatin. Although he had not yet started exercising due to home projects, he remained committed to lifestyle changes. The pharmacist discussed the relationship between cholesterol and lifestyle, including diet and exercise, and suggested switching to atorvastatin 40 mg. The cardiologist initiated 40 mg of atorvastatin, and Peter began this treatment. Seven weeks later, he reported feeling well, had adopted a healthier diet, lost 4 kg, exercise at the gym every other day and achieved an LDL-C of 2.5 mmol/L, a reduction of over 50%. At the final follow-up, two months later, he continued to maintain his healthy lifestyle and lost an additional 5 kg.
Thomas, a 61-year-old man with no prior medical history, experienced a myocardial infarction for which he received a drug-eluting stent. Following the incident, he was diagnosed with diabetes and discharged on ASA, ticagrelor, metoprolol, enalapril, insulin, atorvastatin 40 mg, and later metformin. Four months post-discharge, during a consultation with the pharmacist, he reported persistent fatigue and pain in his legs after walking 200 m, which had prompted thoughts of emergency care. He had been neglecting his atorvastatin for two months, influenced by a friend’s belief that statins cause serious side effects. Although he understood his diabetes well, he found it unsettling that his cardiovascular health metrics were improved yet he felt unwell. The pharmacist explained that his symptoms might indicate claudicatio related to arteriosclerosis and elevated LDL-C. He was advised to control his blood pressure and blood glucose to prevent further complications and encouraged to seek evaluation at his primary care center for LDL-C testing and potential discussions about resuming statin therapy. At a follow-up three weeks later, Thomas remained unchanged in well-being but expressed pride in no longer needing insulin. He had undergone a leg examination at the health center but did not understand the results. The pharmacist clarified that arteriosclerosis had also impacted his legs and discussed the necessity of lowering his LDL-C, now at 3.2 mmol/L (up from 1.8 during treatment). After considering various information from alternative sources, Thomas decided to restart atorvastatin and contemplate quitting oral tobacco. A summary of his personal risk factors and agreed next steps were provided. One month later, he continued taking atorvastatin despite experiencing diffuse symptoms in his extremities and feeling generally weak and fatigued. Thomas had reflected on his health, contemplating depression but attributing his low mood to physical discomfort. Although he had lost appetite, he attributed this to summer heat. The pharmacist ordered a hemoglobin test to rule out anemia. Following a visit with his diabetes nurse, Thomas was relieved to find he could discontinue daily glucose checks. However, he reported persistent lack of appetite and low mood, including some days with suicidal thoughts. Now, after reading materials from his friend linking statins to suicide, he considered stopping atorvastatin again. The pharmacist acknowledged potential links between atorvastatin and depression, advising Thomas to prioritize his mental health while urging him to seek care if suicidal thoughts recurred. Thomas felt secure in his decision to stop atorvastatin and was recommended to inquire about ezetimibe when he felt more stable. At a ten-month follow-up, several medications had been adjusted; Thomas was prescribed ezetimibe but had not initiated it. He had paused enalapril but resumed it regularly due to elevated blood pressure. Now skeptical about pharmacological treatments, particularly lipid-lowering agents, he preferred managing his health with diet while continuing metformin, ASA, clopidogrel, metoprolol, and enalapril. He expressed disinterest in discussions about the risks and benefits of lipid-lowering therapy, prompting the pharmacist to focus on ensuring that Thomas remained stable on his other secondary prevention medications.

### Effect on quality of prescribing

The MAT-CHDsp was applied to EHR data for all study patients six months after inclusion. At this time, the intervention had been initiated for 111 of the 159 patients; therefore, the analysis focuses on these patients. No differences in prescribing quality were found between the intervention and control groups. Detailed results are presented in Table S5 in the Supplement. Overall, adherence to prescription criteria was high (> 80%), while adherence to follow-up criteria was lower. For instance, 80% of patients in both groups were prescribed double anti-platelet therapy (DAPT), with justified non-adherence rates ranging from 12.8% to 16.2% (criteria 2), and approximately 90% of patients with indication were prescribed a beta-receptor blocker (criteria 13–14). In contrast, only about 40% of patients achieved the cholesterol therapy goal (criteria 9), and approximately 50% reached the blood pressure goal (criteria 11). Among patients who did not meet the cholesterol therapy goal (criteria 10), 78.8% in the intervention group vs. 65.5% in the control group (*p* = 0.083) had intensified treatment or documented justification for non-intensification. A similar pattern was observed for therapy adjustment if blood pressure remained above target (criteria 12).

### Changes in beliefs about medicines

The necessity and concerns subscales demonstrated good internal consistency (α = 0.79 to 0.88), aligning with the reliability findings reported by the original developers [19]. At the 15-month follow-up, the BMQ-S data was missing for 18% of patients in the intervention group and 13% in the control group. As previously reported [11], a difference in beliefs was observed between the groups at follow-up; the mean Necessity-Concerns differential (SD) was more positive in the intervention group compared to the control group 7.9 (5.7) vs. 6.3 (5.8), *p* = 0.022.

### Changes in necessity and concern subscales

From baseline to follow-up, there was no change in the mean score of the necessity subscale for either the intervention or control groups; 0.1 (SD 3.1) vs. -0.3 (SD 2.9), *p* = 0.357. However, on the concern subscale, the mean score decreased in both groups, with a more pronounced reduction observed in the intervention group compared to the control group: -1.6 (SD 4.0) vs. -0.6 (SD 3.4) (*p* = 0.025). These findings were consistent in the adjusted analysis (see Supplement for methods). As a result, the NC-differential increased more in the intervention group than in the control group: 1.8 (SD 5.0) vs. 0.2 (SD 4.7), (*p* = 0.011).

### Changes in each item of the scales

On the necessity scale, there were no changes in the mean score for individual items. In contrast, on the concern scale, all items showed a decrease in the mean score (indicating fewer concerns) in the intervention group, whereas this was observed for only two items in the control group. The mean changes from baseline to follow-up for individual items ranged from -0.2 to -0.5 in the intervention group, compared to changes ranging from negligible to -0.2 in the control group. Full details are provided in Table 5.

**Table 5:** Mean changes from baseline for individual items on the BMQ-Specific

Item	Intervention group			Control group		
Necessity scale	mean	SD	*p*	mean	SD	*p*
My health, at present, depends on my medicines	0.0	0.7	0.511	-0.1	0.8	0.302
My life would be impossible without my medicines	0.0	0.9	0.628	-0.1	0.9	0.176
Without my medicines, I would be very ill	0.0	1.0	0.863	-0.0	0.9	0.744
My health in the future will depend on my medicines	0.0	1.0	0.743	-0.1	0.8	0.261
My medicines protect me from becoming worse	0.0	0.8	0.551	-0.1	0.8	0.401
**Concerns scale**						
Having to take medicines worries me	-0.5	1.2	0.000	-0.2	1.2	0.025
I sometimes worry about long-term effects of my medicines	-0.3	1.3	0.004	-0.2	1.0	0.040
My medicines are a mystery to me	-0.2	1.2	0.045	-0.1	1.1	0.572
My medicines disrupt my life	-0.2	1.1	0.052	-0.1	1.0	0.698
I sometimes worry about becoming too dependent on my medicines	-0.4	1.1	0.000	-0.1	1.0	0.156

### Differences between groups in individual items at follow-up

At follow-up, the intervention and control groups differed in their responses to three individual items. The item “My health, at present, depends on my medicines” had a higher mean score (SD) in the intervention group compared to the control group: 4.2 (0.7) vs. 4.0 (0.8), *p* = 0.049. Conversely, two items from the concern scale showed lower mean scores in the intervention group: “Having to take medicines worries me” and “I sometimes worry about becoming too dependent on medicines”: 2.3 (1.2) vs. 2.6 (1.2), *p* = 0.026 and 2.2 (1.1) vs. 2.5 (1.2), *p* = 0.035.

### Details on how patients shifted to or maintained an accepting attitude at follow-up

In most patients, the change in beliefs was insufficient to shift them to a different attitudinal group. Approximately 90% of patients with an accepting attitude at baseline remained accepting at follow-up in both groups (Table S6). Among patients who were ambivalent at baseline, 52.8% of intervention patients vs. 31.0% of control patients (*p* = 0.051) shifted to an accepting attitude.

## Discussion

This study provides insights into the implementation and performance of a complex healthcare intervention aimed at improving medication adherence and achieving cholesterol therapy goals in the secondary prevention of coronary heart disease. The findings from the MIMeRiC study [11] can now be understood through the following key observations from this process evaluation: (1) intervention patients encountered 500 DRPs, the majority of which were handled by pharmacists or prescribers, leading to resolution or improvement in 74% of cases were therapy adjustments were made; (2) no differences in overall prescription quality were observed, (3) the intervention reduced patients’ concerns about their medicines, as intervention patients showed a consistent decline in all concern-related items compared to the control group, and more than half of the intervention patients who were ambivalent at baseline shifted to an accepting attitude.

Regarding intervention fidelity, 144 intervention patients underwent at least one medication review. The high rate of reported DRPs and the significant acceptance of pharmacist suggestions by prescribers indicate strong fidelity to the medication review component of the intervention. The fidelity of MI will be examined in a forthcoming process evaluation study, which will also examine other aspects of the intervention as outlined in the process protocol [13].

The intervention influenced patients’ beliefs, which we hypothesized contributed to improved adherence. This is supported by the fact that all concern items showed a reduction among intervention patients from baseline to follow-up. At follow-up, intervention and control patients in the current study differed on three items that are logically associated with long-term adherence to secondary preventive drugs: “My health, at present, depends on my medicines”, “Having to take medicines worries me”, and “I sometime worry about becoming too dependent on medicines”. While patients with an accepting attitude at baseline remained accepting at follow-up regardless of group, 52.8% of ambivalent patients in the intervention group shifted to an accepting attitude, compared to 31.0% in the control group (*p* = 0.0051). Since a high proportion of patients were already accepting at baseline, there was limited potential for improvement in their beliefs. In a recent study excluding patients who were accepting at baseline, we found that many shifted to a more positive attitudinal category [20]. Although the change in beliefs about medicines in the MIMeRiC trial was modest compared to other studies [21, 22], this process analysis demonstrates that the change was consistently observed across both concern items and patients. We believe the overall change in beliefs is clinically relevant, as it is reflected in the improved adherence observed in the intervention group [11].

The medication reviews of the intervention revealed that nearly all patients had at least one DRP, with most having more than two. These DRPs were either perceived by the patient or identified as unmet clinical targets. Given that patients had received follow-up from both nurses and physicians in standard care, the high number of DRPs may seem surprising. However, reports of suboptimal treatment [23, 24] and patients’ perspectives on medications [25–27] reveal a similar picture. Notably, 67% of patients were documented as experiencing an adverse drug reaction. It is important to note, however, that adverse drug reactions were defined based on patients’ self-reported side effects; sometimes, these reactions were managed through person-centered advice on medication use in daily life.

One strategy to improve overall patient adherence is deprescribing, where discontinuing certain therapies may improve adherence to remaining medications. However, this approach could lead to a decrease in guideline-recommended treatments and may not improve outcomes. In this process evaluation, we aimed to explore whether the quality of treatment was affected by our adherence-improving intervention. Our analysis suggests that deprescribing is unlikely to account for improved adherence in the intervention group, as there were no differences in prescribing patterns between the groups at the 6-month follow-up, and adherence to guidelines remained high. Other registry-based studies have reported high prescription adherence to key therapies, including statins, antiplatelet agents, and ACE/ARB inhibitors [28, 29]. However, the MAT-CHDsp audit tool offers a distinct advantage by incorporating the category of “justified non-adherence”, allowing for more nuanced insights.

Similar to a Norwegian study, we found low adherence to therapy goals for LDL-C and blood pressure, underscoring the challenges both patients face in achieving these goals and the healthcare system faces in supporting them [30]. At the time of our study, evidence-based therapy included simvastatin or atorvastatin, with a transition toward initiating high-intensity doses occurring between 2014 and 2016. In accordance with ESC guidelines [31], patients with statin intolerance were considered for treatment with ezetimibe. However, combination therapy with statins was not recommended until the release of the 2017 ESC guidelines [32] and had not yet become standard practice in Sweden during the MIMeRiC study period [3]. Overall, our study population had lower goal attainment compared to Swedish patients under 75 years old with myocardial infarction in 2019 [28]. This difference may be partly attributed to the temporal gap between the studies, as our study (2014–2018) coincided with earlier, lower rates found in the registry study and included patients over 75. Additionally, our population had a higher proportion of women and patients with angina, both of which are associated with lower likelihoods of achieving LDL-C goals [33].

The MIMeRiC intervention aimed to optimize medication therapy for long-term patient benefit rather than strictly adhering to treatment guidelines. As discussed in the MIMeRiC outcomes paper [11], the seemingly contradictory finding of increased adherence to lipid-lowering treatment without improved and potentially decreased LDL-C goal attainment could be attributed to several reasons. A detailed analysis of pharmacists’ actions on DRPs related to lipid-lowering drugs revealed that therapy was reduced for ten patients. In 35 cases, high LDL-C was accepted for various reasons, often because a re-check of values was suggested, sometimes due to the lack of a more effective treatment option, or because the patient was already on the maximum tolerated dose. Conversely, therapy was intensified for 32 patients due to high LDL-C levels. According to current recommendations, treatment intensification typically involves increasing the statin dose or switching to a more potent statin, such as atorvastatin. A previous analysis demonstrated that adherent intervention patients had lower LDL-C levels than non-adherent patients (2.1 mmol/L vs. 2.5 mmol/L, *p* = 0.049, goal attainment 25.0% vs. 41.8%, *p* = 0.164) [34]. However, the number of patients who achieved adherence through the intervention was insufficient to impact overall LDL-C outcomes in the intervention group. We suggest that the higher adherence without corresponding improvements in LDL-C goal attainment may not be attributable to pharmacists prioritizing long-term treatment strategies, such as agreeing to lower statin doses, as hypothesized in the outcomes paper [11]. A comparison of LDL-C target attainment between the intervention and control groups at baseline (39.6% vs. 41.7% *p* = 0.714 [11]), at six months according to MATCHDsp (40.4% vs. 44.2% *p* = 0.520), and at follow-up (37.0% vs. 44.2% *p* = 0.263 [11]) suggests baseline differences that widened over time. Additionally, the high proportion of missing LDL-C values at follow-up may have contributed to the numerically lower goal attainment observed in the intervention group. Recent Swedish registry studies have found that adherence to lipid-lowering treatment has a greater impact on cardiovascular outcomes than treatment intensity or LDL-C goal attainment [3, 4]. This suggests that the intervention may have long-term benefits for patients’ health. As noted in the outcomes paper [11], the potential long-term benefits of improved adherence on clinical outcomes may take several years to manifest and will be further investigated.

How can we value the intervention in the MIMeRiC study, which improved patients’ beliefs and increased their persistence to secondary preventive medicines through pharmaceutical care? Patients prescribed up to five new medications for a newly diagnosed chronic disease are naturally prone to experiencing DRPs. This process evaluation suggests that medication reviews by clinical pharmacists in the outpatient clinic can improve care, as therapy adjustments were made in 306 cases, and in follow-up, 74% of these changes had resolved or improved the issue. The CATI study in the Netherlands, which targeted the same patient group but in a pharmacy setting, examined a similar intervention [35]. Although 14–32% of their patients expressed negative beliefs about medicines, the intervention activities focused primarily on supporting pill intake and routines, with limited attention to addressing beliefs, and only six attempts to target these beliefs were reported [36]. In contrast, the MIMeRiC intervention placed greater emphasis on addressing beliefs, and the clinical pharmacy setting allowed for more comprehensive management of adverse drug events, including therapy adjustments in response to DRPs. This could explain why our intervention improved adherence, while the CATI study did not. Similarly, the MIRROR trial, conducted by nurses in a cardiovascular outpatient clinic in the Netherlands, did not improve adherence, likely due to its focus on MI without making necessary therapy adjustments [21]. We propose that DRPs are influenced by both beliefs and pharmacological effects, which suggests that interventions should combine therapy adjustments with opportunities for person-centered discussions, such as MI. This approach was also shown in the NAILED-ACS study [37], where nurses at the cardiac clinic conducted follow-up phone calls, discussing and adjusting treatment over an extended period. The results of the MIMeRiC trial and this process analysis suggest that continuous follow-up by a healthcare practitioner who can address treatment experiences and modify prescriptions leads to more personalized treatment. This approach helps patients experience fewer concerns about their medications, thereby increasing adherence.

### Limitations

There are several limitations in this multifaceted process evaluation. First, the report of the medication review process relied on complete documentation by the intervening pharmacists, as the data were retrospectively collected. Additionally, the categorization of DRPs was partly a subjective process and could not be blinded. However, we utilized a recognized categorization framework based on a hierarchical structure and added two additional categories relevant to our specific context. Missing data on the BMQ at follow-up, particularly the higher loss of data among intervention patients, represents another limitation and may have introduced bias in interpreting the findings. Furthermore, the analysis of prescription quality aimed to evaluate treatment quality post-intervention; however, for many patients, the intervention had barely started when the MAT-CHDsp audit tool was applied. Blinding when applying MAT-CHDsp was not feasible, and therefore, observer bias in favor of the intervention group cannot be ruled out. The MAT-CHDsp is an observer-dependent audit tool; however, good inter- and intra-reliability has previously been demonstrated [16, 17]. In this study, a second researcher reviewed a random sample of patient records.

## Conclusions

The MIMeRiC intervention aimed to optimize medication therapy for patients in the long term, rather than strictly adhering to treatment guidelines. This process evaluation demonstrates that this intervention: (1) identified and managed DRPs in collaboration with patients and physicians, with a substantial number being resolved or improved, (2) did not compromise prescription quality, and (3) was effective in reducing patients’ concerns about their cardiovascular medications, as shown by a consistent decline in all concern items among intervention patients. Furthermore, more patients in the intervention group continued using their medicines 15 months after discharge [11]. Collectively, these findings suggest that the intervention may promote sustained adherence to secondary prevention treatment [38]. The question is whether this increased adherence will translate into improved clinical outcomes, despite the lack of improvements in LDL-C goal achievement. As previously reported, no differences in healthcare utilization were observed within the first 15 months [11]. However, secondary prevention treatments require lifelong adherence, and it is anticipated that therapy tailored to patient needs will support sustained adherence and eventually influence healthcare utilization over a longer period. This hypothesis will be further explored in an upcoming three-year follow-up [12].

## Supplementary Information

Below is the link to the electronic supplementary material.

Supplementary Material 1

Supplementary Material 2

## Data Availability

Data cannot be made publicly available to protect participants’ privacy.

## Abbreviations

MIMeRiC: Motivational Interviewing and Medication Review in Coronary Heart Disease
DRP: Drug-Related Problem
CHD: Coronary Heart Disease
MAT-CHDsp: Medication Assessment Tool for Secondary Prevention of Coronary Heart Disease
BMQ-S: Beliefs about Medicines Questionnaire specific
LDL-C: Low-Density Lipoprotein Cholesterol
MI: Motivational Interviewing
EHR: Electronic Health Record
MMAS-8: Morisky 8-item adherence scale
CVD: Cardiovascular Disease
ASA: Acetylsalicylic Acid
HbA1c: Hemoglobin A1c
DAPT: Dual Antiplatelet Therapy
NC-differential: Necessity-Concern differential
ACE-inhibitor: Angiotensin-Converting Enzyme inhibitor
ARB-inhibitor: Angiotensin II Receptor Blocker-inhibitor
RAAS-inhibitor: Renin-Angiotensin-Aldosterone System-inhibitor
LV: Left Ventricle
LVEF: Left Ventricular Ejection Fraction
STEMI: ST-Elevation Myocardial Infarction
NSTEMI: Non-ST-Elevation Myocardial Infarction
RCT: Randomized Controlled Trial
