# Supplementary material for: Impact of a pharmacist adherence intervention on medication beliefs and drug-related problems in coronary patients – a process evaluation of the MIMeRiC trial
Source: BMC Health Serv Res. 2026 Sep 15;26:1283. doi: 10.1186/s12913-026-15037-5 (PMC13589337; doi:10.1186/s12913-026-15037-5)
Supplement: Supplementary file 1 [file 12913_2026_15037_MOESM1_ESM.docx]

Supplement to: **Impact of a** Pharmacist Adherence Intervention on Medication Beliefs and Drug-Related Problems in Coronary Patients – A Process Evaluation of the MIMeRiC Trial

# **Supplementary Methods**

The process evaluation is based on a mix of methods depending on the research question; in detail, this process is as follows:

## Medication reviews

### Data collection

Data on identified drug-related problems (DRPs), actions taken to handle them, and their outcomes were extracted from the pharmacists’ documentation in the electronic health record (EHR) and supplementary study notes that the pharmacists recorded during the intervention. The information from these sources was merged and classified by LH, with any ambiguities discussed and resolved with MJÖ to ensure consensus. Data on actions taken as well as results were also collected from other professionals’ documentation or laboratory data in the EHR.

### Classification of DRPs, actions, and result status

The classification of DRPs is detailed in the main text. Pharmacists’ suggestions, their actions, and physicians’ actions were classified into nine categories: *increasing the dose, decreasing the dose, changing the drug, initiating new drugs, discontinuing drugs, providing patient-specific information/counselling, monitoring or evaluating drug treatment, changing the dosage regimen and no suggestion.*

Results were classified into three status categories (Table S1). If no data were available, the result status was *unknown*. The resulting status was not investigated for two types of DRPs primarily related to the MI component of the intervention: negative beliefs about drugs and insufficient knowledge (as this was both difficult to value and somehow investigated in other ways, i.e., BMQ-S). For these DRPs, the result status was classified as *not applicable*. The *not applicable* category was also used in other cases; see the definitions provided in Table S1.

| **Table S1.** Definitions of result status | |
| --- | --- |
| Result status category | Definition |
| Issue solved | Full resolution of the DRP, such as when side effects are eliminated, or therapy goals are achieved. This also applies if symptoms, previously suspected to be side effects (adverse events), remain unchanged despite discontinuation of the drug. Additionally, issue solved can be considered when a patient fully accepts a suspected or confirmed side-effect. |
| Issue improved | The patient’s status is improved regarding the DRP, although goals are not fully achieved, e.g., a statin dose increase without available information on lipid levels. |
| Issue remains | No improvement in the patient’s status, or the action resulted in new DRPs, leading to unchanged treatment and a stable patient. |
| Unknown | No follow-up data available. |
| Not applicable | When no suggestions have been forwarded.  When suggested actions were not implemented.  When DRPs are no longer present due to other reasons.  When the DRP involves insufficient knowledge or negative beliefs. |
|  |  |

## Quality of prescribing

The Medication Assessment Tool for secondary prevention of coronary heart disease (MAT-CHDsp), originally developed in Norway, was updated by our research group in 2014 to align with newer European, Swedish, and local therapy guidelines. The following are the most important criteria updates: the time for double antiplatelet treatment; a criterion for high bleeding risk added; atorvastatin added to simvastatin as the recommended lipid-lowering therapy; the goal for lipid-lowering therapy changed from <2,5 mmol/L to <1,8 mmol/L; all diabetic patients, regardless of complications, should be prescribed a RAAS inhibitor; only patients with myocardial infarction or left ventricular function <40% are recommended for beta-blocker therapy; and the therapy goal for blood pressure was changed to <140/90 according to Swedish guidelines. The updated MAT-CHDsp was validated in 22 patients in 2014, and most criteria were highly applicable; however, data on lifestyle advice were often missing, and it was suggested that these criteria be excluded because of low applicability and because they are not related to drug therapy. In 2018, the updated MAT-CHDsp was assessed against newer guidelines and found to remain valid for the study period (2013—2016). The only amendment in 2018 was the exclusion of the four lifestyle criteria. AAM used the MAT-CHDsp to assess treatment quality by retrospectively reviewing the EHR for prescribed medications, diagnoses, physicians’ and pharmacists’ documentation of possible side effects or justifications for treatment choices, and selected laboratory parameters for the period between inclusion and 6 months. MJÖ validated the application of the MAT-CHDsp in 20 study patients.

For each applicable criterion, the reviewer recorded *yes*, *no but justified*, *no*, or *missing data*. Adherence to guidelines was assessed based on all *yes* of all applicable cases. Additionally, the MAT-CHDsp provides a measure of justified nonadherence, where guidelines were not followed due to a documented, justified reason in the EHR. The results are reported as proportion adherence and proportion justified nonadherence, with groups compared across these categories (i.e., guideline-recommended *or* justified treatment versus nonadherence). As adherence, justified nonadherence, and nonadherence are categorical variables, the chi-square test was used, and Fischer’s exact test was applied where the assumptions for the chi-square test were not met. Missing data (8 criteria applications in the intervention group and 16 in the control group) were excluded from the analysis.

**Figure S1.** Copy of the assessment tool used, MAT-CHDsp amended version based on the original version developed by Garcia et al. [1]

## Details about BMQ-S assessment

Data on beliefs about medicines were collected in the MIMeRiC trial using the Beliefs About Medicines Questionnaire Specific (BMQ-S), administered at baseline (after standard care follow-up), after 10 months (during the intervention), and after 15 months (post- intervention). The BMQ-S consists of two subscales: one for the perceived necessity of the drugs and one for perceived concerns about the drugs. Each subscale contains five items, assessed on a five-point verbal descriptor scale ranging from “strongly disagree” to “strongly agree.”

As instructed by the originators, numerals (1-5) were assigned to each statement of agreement to summarize each subscale, yielding a score range of 5-25 points per subscale. The difference between the necessity and concern scales known as the Necessity-Concerns differential (range -20 to +20) reflects the individual patient’s cost‒benefit analysis of taking their medicines. A positive differential indicates that the patient perceives the benefit of taking the drugs to outweigh the risks, and the more positive the difference, the higher the likelihood of adherence [2]. It has also been found that categorizing patients based on high (>15) or low (<15) scores on each scale, resulting in four categories (accepting, ambivalent, neutral, and skeptical), can predict adherence behavior [3, 4].

# **Supplementary Results**

## Actions and results of medication reviews

| **Table S2.** Suggested and performed actions to DRPs in medication reviews | | |
| --- | --- | --- |
| **Suggested actions to DRPs** | **Number of suggested actions for each type (percentage of total number of suggested actions)** | **Performed actions (percentage of suggested actions)** |
| Increase dose | 41 (8%) | 37 (90%) |
| Decrease dose | 31 (6%) | 28 (90%) |
| Change drug | 45 (9%) | 42 (91%) |
| Initiate new drug | 30 (6%) | 22 (73%) |
| Discontinue drug | 46 (9%) | 36 (78%) |
| Patient-specific information/counselling | 169 (34%) | 169 (100%) |
| Monitor or evaluate drug treatment | 88 (18%) | 52 (63%) |
| Change in dosage regimen | 26 (5%) | 21 (80%) |
| No suggestion | 24 (5%) | not relevant |
| Total | 500 (100) | 405 (80%) |

| **Table S3.** Communication methods and corresponding prescriber actions | | |
| --- | --- | --- |
| **Communication method** | **Number of suggestions communicated this way** | **Number of actions by prescriber (percentage of suggested actions)** |
| Face-to-face | 76 | 66 (87%) |
| Messenger in EHR | 36 | 31 (86%) |
| Noted in the EHR | 4 | 4 (100%) |
| Referral | 38 | 27 (71%) |
| Unknown | 20 | 18 (90%) |

**Figure S2**. Management of DRPs related to lipid-lowering drugs.

DRPs for lipid-lowering drugs could either be related to a need for more treatment or may require a decrease or change in treatment. Some DRPs were related to nonadherence, knowledge, or beliefs and were managed through counselling only.

Following the path of potentially needing more treatment, the pharmacists either had no suggestion, had a suggestion but not to change treatment, or suggested increasing the treatment. These suggested actions were either performed or not, and for those that were performed, the figure also illustrates the results of this management.

Table S4 (attached) summarizes nine patient cases, illustrating how medication reviews were conducted regarding lipid-lowering treatment and their corresponding outcomes.

The proportions of adherence and justified nonadherence to the criteria are calculated based on applicable cases for each criterion. Applicable cases refer to cases where a specific criterion is relevant to the patient, including those where MAT-CHDsp could not be applied due to missing data.

^1^Chi2 test 2x2 for adherent and justified nonadherent versus nonadherent. ^2^not testable, for example “0” in one of the columns in the crosstabulation. ^3^Fischer’s exact test.

## Belief changes

Among the 58 patients in the intervention group who were accepting at baseline, 53 (91.4%) were still accepting at follow-up, and the pattern was similar in the control group: of 74 accepting at baseline, 66 (89.2%) were still accepting at follow-up. Among patients who were ambivalent at baseline (36 intervention patients and 42 control patients), 52.8 % and 31.0 % (p=0.051) had changed their attitudes to accepting at follow-up.

| **Table S6**. Accepting attitude at follow-up based on attitude at baseline and group, PP sample | | | | | | | |
| --- | --- | --- | --- | --- | --- | --- | --- |
|  | **Accepting at follow-up** | | | | |  | |
| **Attitude at baseline** | **Intervention** | |  | **Control** | |  |  |
|  | **n** | **%** |  | **n** | **%** |  | ***p*** |
| Accepting (58 I, 74 C) | 53 | 91.4 |  | 66 | 89.2 |  | *0.675* |
| Ambivalent (36 I, 42 C) | 19 | 52.8 |  | 13 | 31.0 |  | *0.051* |
| Indifferent (8 I, 5 C) | 3 | 37.5 |  | 4 | 80.0 |  | *na* |
| Skeptical (6 I, 13 C) | 2 | 33.3 |  | 5 | 38.5 |  | *na* |

Abbreviations: I, Intervention; C, Control; na, Not Applicable

# **References**

1.	Garcia BH, Utnes J, Naalsund LU, Giverhaug T: **MAT-CHDSP, a novel medication assessment tool for evaluation of secondary prevention of coronary heart disease**. *Pharmacoepidemiology and drug safety* 2011, **20**(3):249-257.

2.	Horne R, Weinman J: **Patients' beliefs about prescribed medicines and their role in adherence to treatment in chronic physical illness**. *Journal of psychosomatic research* 1999, **47**(6):555-567.

3.	Horne R, Parham R, Driscoll R, Robinson A: **Patients' attitudes to medicines and adherence to maintenance treatment in inflammatory bowel disease**. *Inflamm Bowel Dis* 2009, **15**(6):837-844.

4.	Tibaldi G, Clatworthy J, Torchio E, Argentero P, Munizza C, Horne R: **The utility of the Necessity--Concerns Framework in explaining treatment non-adherence in four chronic illness groups in Italy**. *Chronic illness* 2009, **5**(2):129-133.
