# Supplementary material for: Impact of a pharmacist adherence intervention on medication beliefs and drug-related problems in coronary patients – a process evaluation of the MIMeRiC trial
Source: BMC Health Serv Res. 2026 Sep 15;26:1283. doi: 10.1186/s12913-026-15037-5 (PMC13589337; doi:10.1186/s12913-026-15037-5)
Supplement: Supplementary file 2 [file 12913_2026_15037_MOESM2_ESM.docx]

| **Table S4**. A summary of nine patient cases illustrating how medication reviews were conducted for cholesterol treatment (white columns) and corresponding data for selected process measures and outcomes in the MIMeRiC trial (grey columns). | | | | | | | | | |
| --- | --- | --- | --- | --- | --- | --- | --- | --- | --- |
| **Patient** | **Issue** | **Reasoning** | **Result** | **DRP** | | **BMQ-Specific** | | **LDL-C outcome in RCT** | **Adherence outcome in RCT** |
|  |  |  |  | **Type** | **Outcome** | **Before** | **After** |  |  |
|  |  |  |  |  |  |  |  |  |  |
| 77-year-old man with STEMI treated with DES | At the medication review, the patient is only on 10 mg of Atorvastatin, which is not a high-potency dose. | Dose too low, but this is due to earlier nausea related to the 40 mg statin. | No adjustment is made; at follow-up, the patient has 10 mg and LDL-C 1.9 mmol/L | Dosage too low | NA | Ambivalent  Necessity 18  Concern 15 | Accepting  Necessity 23 Concern 13 | 1.9  Fail | Adherent |
|  |  |  |  |  |  |  |  |  |  |
| 74-year-old woman with STEMI treated with DES | Stomach pain and reflux with Atorvastatin 80 mg. Initial value 4.5 mmol/L and with 80 mg 3.5 mmol/L. | Suspected adverse events, decrease dose. | Dose decreased to 40 mg and tolerated, later also tried increasing to 60 mg but intolerable. | Adverse drug reaction | Improved | Accepting  Necessity 19 Concern 8 | Accepting  Necessity 18 Concern 10 | Missing | Adherent |
|  |  |  |  |  |  |  |  |  |  |
| 66-year-old man with NSTEMI treated with DES | Atorvastatin 40 mg and 1.8 mmol/L, which is just above the treatment target. | Dose might be too low, re-evaluate. | LDL-C increases to 2.2 but ApoB is 0.66 and no other risk-factors. Treatment is left unchanged. | Dosage too low | Solved | Ambivalent  Necessity 25 Concern 20 | Ambivalent  Necessity 16 Concern 19 | LDL 2.2, Fail* | Adherent |
| 69-year-old man with CCS treated with DES | LDL-C is 3.1 mmol/L. on pravastatin 40 mg. | LDL-C should be lowered, but the patient has had statin intolerance with other statins. Other DRPs concerning diabetes and BP are prioritized. No statin change suggested. Detailed description in Box 1. | At 10-month, diabetes and BP controlled, LDL-C improved but 2.3 mmol/L. No change suggested due to prior intolerance. | Ineffective drug | NA | Accepting  Necessity 25 Concern 10 | Accepting  Necessity 25 Concern 10 | 2.4  Fail | Adherent |
| 61-year-old man with STEMI, HF, and pericarditis, as well as DM2 in need of insulin | At first study visit (4 months after discharge), patient stopped Atorvastatin 40 mg due to a good value; 1.8 mmol/L (initial 2.7) and because a friend has informed him about statins being deadly. Patient feels tired unwell and has recently experienced problems when walking longer distances that appear as claudication. | Discussion with patient and recommendation to control LDL-C again, and referral to GP for suspected claudicatio.  At a new visit 3 weeks later, he is still tired, LDL-C 3.5 mmol/L, diagnosed with claudication. Patient agrees to try Atorvastatin again. Receives a personal risk factor summary and how he could manage them. One month later, he is still taking Atorvastatin and intent on continuing. He is fatigued with no appetite, has considered depression. He is controlled for anemia and scheduled for a new follow-up.  Two weeks later, he reports that he has been really low a few days earlier and even had suicidal thoughts. He has now read that statins have been linked to suicide, so he thinks he’d better stop Atorvastatin again. Detailed description in Box 1. | Patient decides finally to stop Atorvastatin. Agrees with pharmacist to contact his GP for a prescription of ezetimibe when ready and seek support if suicidal thoughts persist.  4 months later: prescribed ezetimibe by his GP but not taking it. Resumed enalapril after a pause, and continues metoprolol, metformin, aspirin, and ticagrelor, with no intention to stop them. | Nonadherence  Nonadherence | Improved  NA | Accepting  Necessity 18 Concern 10 | unknown | Missing | Nonadherent |
| 69-year-old man with STEMI treated with DES | LDL-C 2.0 mmol/L on Atorvastatin 40 mg. Initial value 3.5 mmol/L. | Dose too low; increase could be beneficial, but physician had already informed the patient that the dose should be re-evaluated | No dose-adjustment. | Dosage too low | NA | Ambivalent  Necessity 22 Concern 19 | Ambivalent  Necessity 20 Concern 19 | 1.6  Success | Adherent |
| 67-year-old man with earlier NSTEMI who had progression of atherosclerosis treated with DES | LDL-C 3.8 mmol/ L at discharge, lowered to 2.1 mmol/L with Atorvastatin 40 mg. | Treatment intensified, in particular due to atherosclerosis progression. Patient agrees to increase Atorvastatin to 60 mg. However, the patient experiences difficulty remembering to take it at night and is advised to switch to morning intake. | LDL-C lowered to 1.4 mmol/L, and patient is satisfied. At the last follow-up (10m months), patient becomes more ambivalent after reading about statins and possible side effects and questions the need for the high dose. Discussion at this meeting ends with an acceptance for now. | Dosage too low  Nonadherence | Solved  Solved | Ambivalent  Necessity 19 Concern 16 | Accepting  Necessity 21 Concern 14 | LDL-C 1.6 mmol/L  Success | Adherent |
|  |  |  |  |  |  |  |  |  |  |
| 74-year-old woman with previous CHD presenting with unstable angina, treated with DES | Initial Atorvastatin dose was lowered to 20 mg due of unspecific bowel issues. LDL-C initially 3.6 and now 2.1 on 40 mg Atorvastatin. | Dose too low; an increase could be beneficial. Patient has not experienced improvements in bowel issues since dose reduction. Detailed description in Box 1. | Dose increased; new LDL-C 1.8 mmol/L. No further adjustment needed. | Dosage too low | Improved | Accepting  Necessity 21 Concern 11 | Ambivalent  Necessity 21 Concern 15 | Missing | Adherent |
| 69-year-old man with CCA treated with DES | LDL-C decreased from 5.6 to 3.2 mmol/L with Simvastatin 40 mg. | Potent statin needed. | Changed to Atorvastatin 40mg. | Ineffective drug | Solved | Ambivalent  Necessity 18 Concern 16 | unknown | 2.2 = 50% decrease, Success | Adherent* |

Abbreviations: BMQ-Specific, Beliefs about Mediicines Questionnaire Specific; BP, Blood pressure; CCA, Chronic coronary syndrome; CHD, Coronary heart Disease; DES, Drug-eluting stent; DM2 Diabetes mellitus type 2; DRP, Drug related problem; GP, General practitioner; HF, Heart failure; LDL-C, Low-density lipoprotein cholesterol; NA, Not applicable; NSTEMI, Non-ST-elevation myocardial infarction; RCT, Randomized controlled trial, in this case MIMeRiC; STEMI, ST-elevation myocardial infarction

*Adherent according to medication refill but self-report questionnaire not submitted.
